# Effects of a Maternal Essential Fatty Acid and Conjugated Linoleic Acid Supplementation during Late Pregnancy and Early Lactation on Hematologic and Immunological Traits and the Oxidative and Anti-Oxidative Status in Blood Plasma of Neonatal Calves

**DOI:** 10.3390/ani11082168

**Published:** 2021-07-22

**Authors:** Wendy Liermann, Katrin Lena Uken, Christine Schäff, Laura Vogel, Martina Gnott, Armin Tuchscherer, Erminio Trevisi, Tadeusz Stefaniak, Helga Sauerwein, Arnulf Tröscher, Harald Michael Hammon

**Affiliations:** 1 Institute of Nutritional Physiology “Oskar Kellner”, Leibniz Institute for Farm Animal Biology (FBN), 18196 Dummerstorf, Germany; liermann@fbn-dummerstorf.de (W.L.); uken@fbn-dummerstorf.de (K.L.U.); cschaeff@web.de (C.S.); vogel@fbn-dummerstorf.de (L.V.); gnott@fbn-dummerstorf.de (M.G.); 2 Institute of Genetics and Biometry, FBN, 18196 Dummerstorf, Germany; tuchscherer.dummerstorf@t-online.de; 3 Department of Animal Sciences, Food and Nutrition (DIANA), Università Cattolica del Sacro Cuore, 29122 Piacenza, Italy; erminio.trevisi@unicatt.it; 4 Department of Immunology, Pathophysiology and Veterinary Preventive Medicine, Faculty of Veterinary Medicine, Wroclaw University of Environmental and Life Science, 50-375 Wroclaw, Poland; tadeusz.stefaniak@upwr.edu.pl; 5 Institute of Animal Science, Physiology and Hygiene Unit, University of Bonn, 53115 Bonn, Germany; Sauerwein@uni-bonn.de; 6 BASF SE, 68623 Lampertheim, Germany; arnulf.troescher@basf.com

**Keywords:** calf, essential fatty acid, conjugated linoleic acid, inflammation, anti-oxidants, bilirubin

## Abstract

**Simple Summary:**

Fatty acids play an important role in the regulation of inflammation and oxidative stress. The birth and the neonatal period are characterized by a high risk of inflammation and an increased production of reactive oxygen species in the calf. The present study deals with the effects of a different maternal fatty acid supply including the supplementation of saturated fatty acids by coconut oil, essential fatty acids, conjugated linoleic acid or a combination of essential fatty acids and conjugated linoleic acid on the immunological and oxidative as well as anti-oxidative status of neonatal calves. Maternal essential fatty acid as well as conjugated linoleic acid supply affected the inflammatory response and the oxidative and anti-oxidative status of the neonatal offspring. Essential fatty acids might have beneficial effects on the prevention of dysregulated inflammation after birth and reduced the plasma bilirubin concentrations in this period. Conjugated linoleic acid and saturated fatty acids might increase the inflammatory response. Similarly, plasma bilirubin increased, which in part might serve as a protector against oxidative stress in the early phase after birth.

**Abstract:**

Fatty acids are known for their regulatory role in inflammation and oxidative stress. The present study investigated 38 calves born from dams, abomasally supplemented with coconut oil, essential fatty acids (EFA), conjugated linoleic acid (CLA) or EFA + CLA, according to immunological traits and the oxidative and anti-oxidative status for the first 5 days of life. On day 2 of life, plasma total bilirubin, cholesterol, interleukin 1-β and ferric ion reducing anti-oxygen power (FRAP) were lower in calves with than without maternal EFA supplementation, and FRAP additionally on day 4. On day 3, the concentrations of reactive oxygen metabolites were higher in calves with than without maternal EFA supplementation and additionally on day 5 together of retinol. Total leucocyte counts were decreased in the EFA group compared to the CLA group on day 5. Lymphocyte proportions decreased from day 1 to 5 only in the EFA + CLA group. On day 2, plasma total protein was higher in CLA and EFA + CLA than in EFA calves. Similarly, CLA calves had higher interleukin 1-β concentrations compared to EFA + CLA calves. FRAP was decreased by CLA on day 4. Overall, the maternal fatty acid supply affected the inflammatory response and the oxidative and anti-oxidative status of the neonatal offspring.

## 1. Introduction

Neonatal calves are at risk of sickness because of their immature immune system and the elevated stress level due to the birth process itself and the exposure to an oxygen-rich environment leading to an increased generation of reactive oxygen species [1,2]. Oxidative stress is defined as an imbalance between the oxidative and the anti-oxidative system of the organism, characterized by an excessive production of reactive oxygen species and the incapacity to neutralize them, leading to cellular DNA, protein and lipid damage [3,4]. Providing the newborn with adequate colostrum intake immediately after birth is essential for reducing calf mortality and morbidity [5,6]. Metabolic stress in dairy cows leads to lipid mobilization, inflammation and oxidative stress and metabolic stress in the prenatal period, which has an impact on the metabolic and inflammatory response of calves [7,8,9,10,11]. Thus, an elevated maternal oxidant status index increased serum concentrations of reactive oxygen and nitrogen species in calves [8].

Beside immunoglobulins, immunomodulatory factors and anti-oxidative substances, colostrum contains long-chain fatty acids that may play a crucial role in inflammation and immune regulation and affect the oxidative status of neonatal calves [12]. In studies of Trevisi et al. [7], it was shown that a supplementation of n-3 fatty acids in the transition period mitigates the impact of subclinical inflammation and has beneficial effects on the energy balance of cows. An influence of maternal fatty acid supplementation on the neonatal immunoglobulin and inflammatory as well as anti-oxidative status was shown in a previous study of Garcia et al. [13] in calves. The supplementation of n-3 fatty acid by milk replacer affected phagocytosis of monocytes, neutrophil oxidative burst capacity and the secondary humoral response in calves [14]. Furthermore, maternal fatty acid supplementation and especially dietary n-6 to n-3 fatty acid ratio can influence the immunoglobulin and cytokine production in piglets [15].

Conjugated linoleic acids (CLA) are produced by the natural biosynthesis of unsaturated fatty acids in the rumen of cows and also enter the milk in higher concentrations [16]. They are involved in cytokine modulation and immunoglobulin synthesis; they are able to influence the anti-oxidative status and the proliferation of bovine peripheral blood mononuclear cells (PBMC) [16,17,18]. In cows, CLA influenced the fibrinogen release in the transition period [19]. Furthermore, CLA have inhibitory effects on the production of reactive oxygen species and the transcription of pro-inflammatory cytokines in bovine mammary epithelial cells [20]. The knowledge of the effects of these fatty acids on the inflammatory and oxidative as well as anti-oxidative status in calves is scanty. However, studies of Dänicke et al. [21] gave evidence that stimulation ability of PBMCs in calves is influenced by maternal CLA supplementation.

Elevated n-3 FA and CLA concentrations are known from cows on pasture or fed fresh grass [22,23,24]. During gestation and via the intake of colostrum and milk, the maternal supply of essential fatty acids (EFA) can be transferred to the calf [13,25]. The first results of the present study showed increased n-3 fatty acid and CLA concentrations, as well as a decreased n-6: n-3 fatty acid status in colostrum and in the blood plasma of calves when dams were supplemented with EFA (mainly n-3 fatty acids provided by linseed oil) and CLA (*cis*-9, *trans*-11 CLA and *trans*-10, *cis*-12 CLA) during late gestation and early lactation [26,27]. Therefore, the present study aimed to investigate the inflammatory and oxidative status as well as anti-oxidative response of calves born from cows, supplemented abomasally with coconut oil as a saturated fatty acid source, EFA and CLA during late pregnancy and early lactation, within the first 5 days after birth. We hypothesized that a combined maternal EFA and CLA supply would promote the immune as well as the inflammatory and anti-oxidative status in calves immediately after birth.

## 2. Material and Methods

The current study was conducted at the experimental station of the Leibniz Institute for Farm Animal Biology (FBN), Dummerstorf from December 2015 to September 2017, in accordance with the guidelines of the German Animal Protection Law. The housing conditions and experimental procedures were approved by the Landesamt für Landwirtschaft Lebensmittelsicherheit und Fischerei Mecklenburg-Vorpommern, Rostock, Germany (registration number 7221.3-1-052/15).

### 2.1. Animals, Experimental Design, and Husbandry

In total 38 Holstein calves (14 male calves, 23 female calves; birth weight: 42.1 ± 4.9 (means ± SD)) were investigated during the first 5 days of life in the current experiment. The calves were born from 37 Holstein cows (36 single born calves and 1 twin pair), which were fed a total mixed ratio with corn silage as main forage component. Cows were abomasally supplemented with coconut oil (control group, CON), essential fatty acids (EFA), CLA or a combination of essential fatty acids and conjugated linoleic acid (EFA + CLA) from d 63 before calving until early lactation (week 9 postpartum; 2nd to 3rd lactation) as represented in Table 1 and described in detail in the study of Vogel et al. [27]. For technical reasons, the study was subdivided into 5 consecutive blocks including 7−8 calves born per block.

Immediately after birth, calves were separated from their dams, weighed, and subsequently supplied with first colostrum, which was fed within the first 2.5 ± 1.7 h of life. Within the first 24 h calves received an amount of 10% of body weight (BW) (provided in two meals). The feed allowance on day 2 of life amounted to only 6% of BW to ensure that all calves received the same amount of colostrum and transition milk during the first 48 h of life irrespective of their birth time. From day 3 onwards, calves were fed an amount of 12% of BW per day, which was provided in 2 meals. Details on feeding management including the milk composition were recently published in a companion paper [26].

In general, colostrum and transition milk fed to the calves were derived from their own dam. The first 2 meals consisted of first colostrum. If the quantity of first colostrum was insufficient, required amounts were complemented by milk from the second milking after parturition. In the following days, the morning milking, was fed to the calves, respectively. If the morning milking was insufficient, the milk gained from the evening milking was also considered. Only if colostrum and transition milk quantity of a dam was insufficient, colostrum or transition milk from another cow of the respective group was added [26]. Calves were fed by nipple bottle and refused milk was tube fed. All calves had free access to water. During the whole experiment, calves were housed individually in boxes, which were integrated in a climate-controlled room (19 °C) and littered with chopped straw.

Calves were monitored daily to determine their general health status including the determination of body temperature and macroscopic assessment of feces. Two calves of the CON group, one calf of the EFA group and one calf of the EFA + CLA group showed a temporary elevated body temperature (≥39.6 °C). Four animals (CON = 2 calves, EFA = 1 calf, EFA + CLA = 1 calf) transiently suffered from diarrhea. Calves were medically treated according to the care instructions of the veterinarian (CON = one calf with analgesic and antibiotic agent; 1 calf with antibiotic and 2 calves with analgesic agent; CLA = one calf with analgesic).

### 2.2. Sample Collection

Samples of first colostrum and subsequent transition milk as fed to the calves were taken and stored at −20 °C until further analyses. Blood samples were taken before colostrum feeding and daily before the morning feeding from the jugular vein, using commercial K_3_EDTA (day 1–2: 9 mL; day 3–5: 4.9 mL), lithium heparin (2 mL) and sodium fluoride/potassium oxalate (2 mL) tubes (1.2–2 mg of K_3_EDTA/mL; 12–13 IU heparin/mL; 2–4 mg/L sodium fluoride and 1–3 mg/L potassium oxalate; Greiner Bio-One International GmbH, Kremsmünster, Austria). Blood samples were placed on ice before centrifugation at 2700× *g* and 4 °C for 20 min. The obtained plasma samples were stored at −20 °C until analyses.

### 2.3. Milk Analyses

Thawed milk samples were homogenized by warming (41 °C) and pivoting before further analyses.

Concentrations of immunoglobulin (Ig) G1, IgG2, and IgM in milk were determined by ELISA as previously described by Gerbert et al. [28]. The intra-assay coefficients of variation (CV) were 9.9, 5.5, and 8.2% and the inter-assay CV were 2.7, 10.6, and 7.1% for IgG1, IgG2, and IgM, respectively. The Ig concentrations in milk (wt/wt) were computed by correction for the density of milk from the respective milking according to data from Madsen et al. [29].

### 2.4. Blood Analyses

The hematocrit and hemoglobin concentration in whole blood as well as the specific blood cell traits (total counts of erythrocytes, red cell distribution width (RDW), mean corpuscular volume of erythrocytes (MVC), mean corpuscular hemoglobin of erythrocytes (MCH), mean corpuscular hemoglobin concentration of erythrocytes (MCHC), total counts of leucocytes, proportions of atypical lymphocytes, proportions of basophilic granulocytes, proportions of thrombocytes, mean thrombocyte volume (MTV), thrombocrit, and immature cells) were determined by an automatic hematology analyzer (ABX Pentra 60, Horiba ABX SAS, Montpellier, France).

Plasma IgG was analyzed in diluted lithium heparin plasma (1:300,000) using a bovine specific sandwich ELISA (#E10-118; Bethyl Laboratories Inc., Montgomery, TX, USA). Absorbance was measured at 450 nm with a multi-detection microplate reader (BioTek Synergy 2, Winooski, VT, USA) and was analyzed with Gen5 software (BioTek). The intra- and inter-assay CV for IgG measurement were 8 and 12%, respectively.

Sodium fluoride/potassium oxalate containing plasma was analyzed for total protein, albumin, cholesterol and total bilirubin using an automatic spectrophotometer (ABX Pentra 400, Horiba ABX SAS) and the following kits: total protein (#553–412) and cholesterol (#553-126) from mti-diagnostics (Idstein, Germany); albumin (#A11A01664) and total bilirubin (#LT-BR 0500) from LABOR + TECHNIK Eberhard Lehmann GmbH, Berlin, Germany).

Haptoglobin was analyzed in K_3_EDTA plasma by ELISA as published by Hiss et al. [30] with an intra- and inter-assay CV of 9.5 and 11.8%.

Interleukins were measured in twofold diluted lithium heparin plasma using bovine-specific commercially available colorimetric sandwich ELISA kits for interleukin 1-β (Cat. No. ESS0027; Thermo Scientific, Frederick, MD, USA) and interleukin 6 (Cat. No. ESS0029; Thermo Scientific). The intra- and inter-assay CV were 4.5 and 17.0% for interleukin 1-β and 3.5 and 13.4% for interleukin 6.

Concentrations of retinol, β-carotene and tocopherol were determined in lithium heparin plasma after extraction with hexane by reverse-phase HPLC (LC4000; JASCO Europe Ltd., Cremella, LC, Italy) with a Zorbax Eclipse Plus C18 column (150 × 4.6 mm, 3.5 µm; Agilent Technologies, Santa Clara, CA, USA), an UV detector set at 325 nm (for retinol), 460 nm (for β-carotene), and 290 nm (for tocopherol), using methanol: tetrahydrofuran (80:20) as mobile phase. A spectrophotometric method modified by Regenhard et al. [31] was used to determine derivatives of reactive oxygen metabolites (dROM) in K_3_EDTA plasma. Intra- and inter-assay CV were 6.3 and 10.0%, respectively. The ferric ion reducing antioxidant power (FRAP) in K3EDTA plasma was analyzed according to Benzie and Strain [32] with intra- and inter-assay CV of 2.7 and 2.6%, respectively. Oxygen radical absorbance capacity (ORAC) was measured in lithium heparin plasma according to the procedure of Cao and Prior [33].

### 2.5. Statistical Analyses

For statistical analyses the MIXED procedure of SAS 9.4 for Windows (SAS Institute Inc., Cary, NC, USA) was used. The model included the fixed effects of the maternal fatty acid supplementation of EFA (level: yes, no), CLA (levels: yes, no), time (levels: day relative to birth) and their interactions. Furthermore, the block and the sex of the calf were considered as fixed factors. The duration of the maternal supplementation and gestation length were added as covariates and the calf was considered as subject for the repeated factor time. The Tukey–Kramer test was used to analyze pairwise differences of least-squares means (LSMeans). The SLICE statement of the MIXED procedure was considered for the separated analyses of LSMeans for interactions. Effects were defined as significant if *p* < 0.05. Pearson correlations according were determined by the CORR procedure of SAS 9.4 and were assessed as significant if *p* < 0.05 and if the correlation power > 0.8. The correlation power was determined by the POWER procedure of SAS 9.4.

## 3. Results

None of the analyzed milk or blood parameter was affected by sex (*p* > 0.05). Therefore, sex will not be further considered in the following descriptions and discussions.

### 3.1. Hematology

While the values of hematocrit, hemoglobin, erythrocyte cell counts, MCV and number of atypical lymphocytes decreased with age, the MCHC and basophilic granulocytes increased with increasing age in all calves (*p* < 0.05; Table 2). Total cell counts of leucocytes were influenced by EFA and time (*p* < 0.05). EFA and EFA + CLA calves showed lower total leucocyte counts on day 5 of life compared to CLA calves (*p* < 0.05). Proportions of lymphocytes decreased with time after birth in all calves except for EFA calves, which were slightly increased from day 1 to 5 of life. A significant CLA-by-time-interaction was detected in case of lymphocyte proportions (*p* = 0.029).

### 3.2. Immunoglobulins in Colostrum and Transition Milk

**Figure 1:**
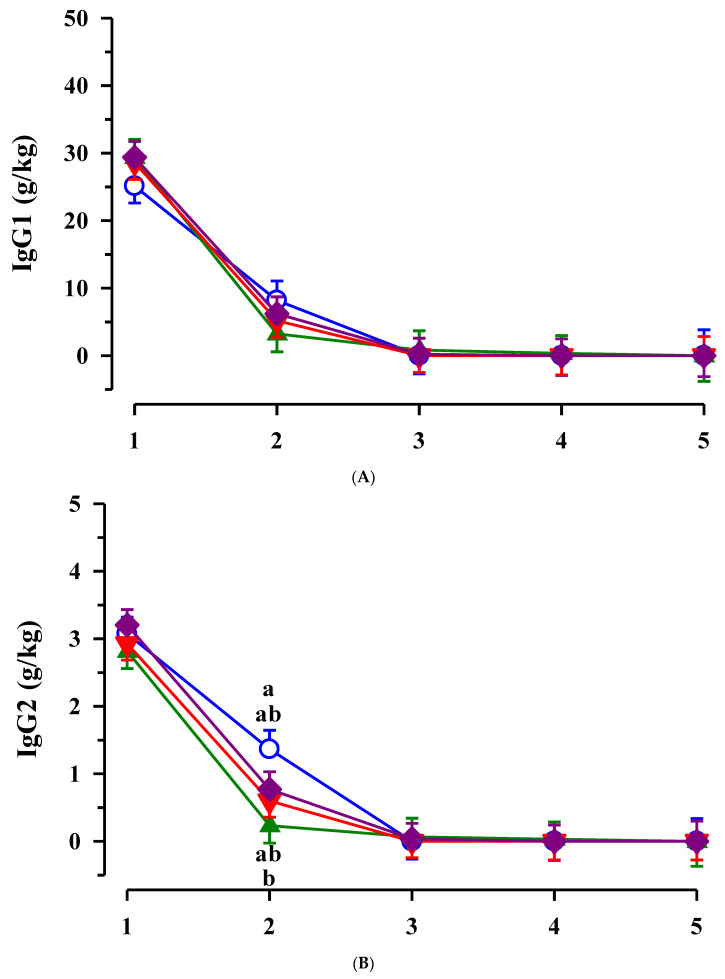

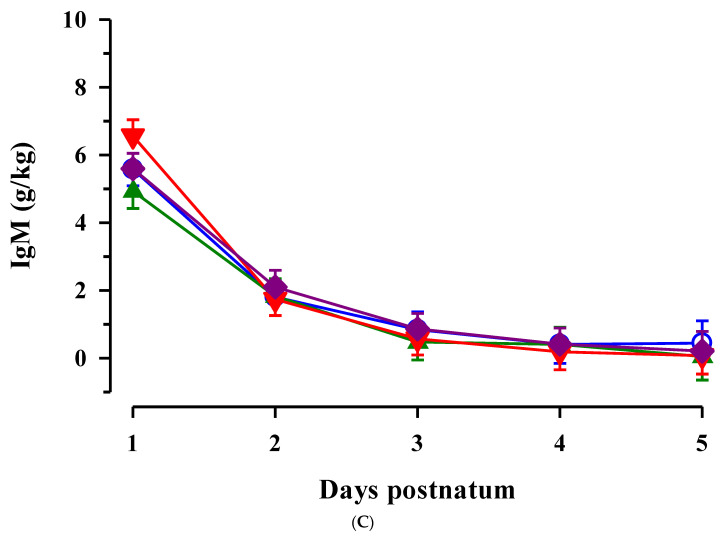
Immunoglobulin (Ig) G_1_ (**A**), IgG_2_ (**B**) and IgM (**C**) concentration in colostrum and transition milk of cows receiving fatty acid supplementation (○ control, CON *n* = 9; ▲ EFA, essential fatty acids, *n* = 9; ▼, CLA, conjugated linoleic acid, *n* = 9; ♦, EFA + CLA, *n* = 11) and time after calving. Day 1 correspond to the day of birth. ^ab^ Different superscripts mark significant differences between groups at similar time points (*p* < 0.05).

The concentrations of IgG_1_ and IgM decreased in colostrum and transition milk after calving (*p* < 0.05) but did not differ between groups with different maternal fatty acid supply (Figure 1A,C). Colostral IgG_2_ concentrations decreased after calving as well but the decrease was slower in CON cows leading to a higher IgG_2_ concentration in transition milk of CON cows compared to EFA cows on day 2 after calving (*p* < 0.05; Figure 1B).

### 3.3. Immunoglobulin G, Metabolites, Interleukins, Vitamins and Oxidative and Anti-Oxidative Traits in Plasma of Calves

**Figure 2:**
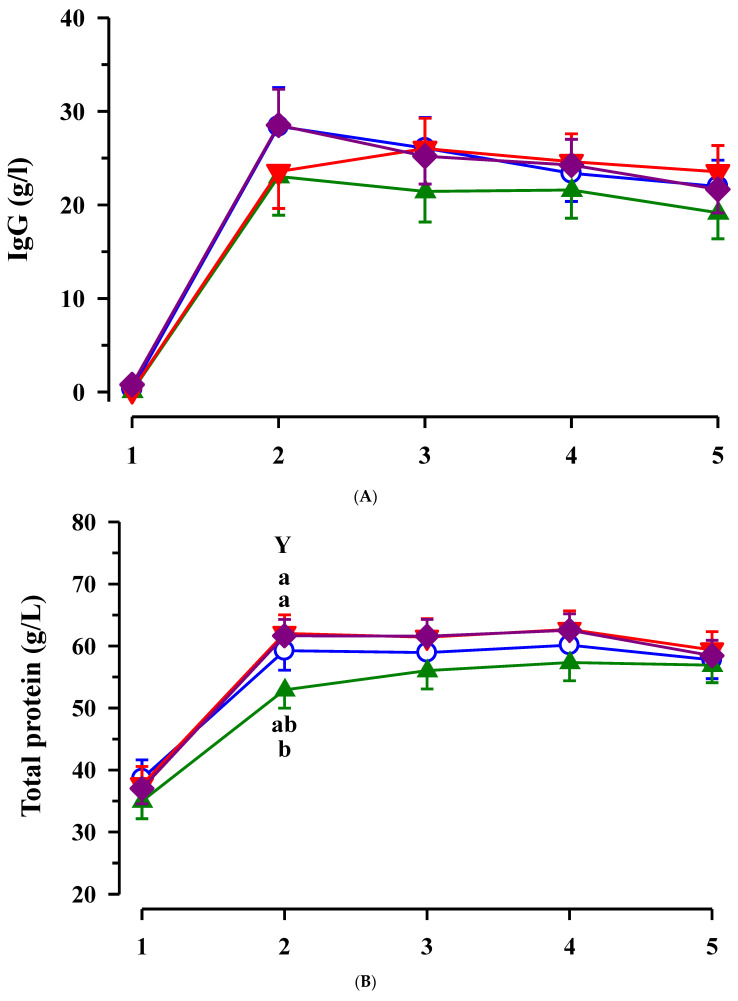

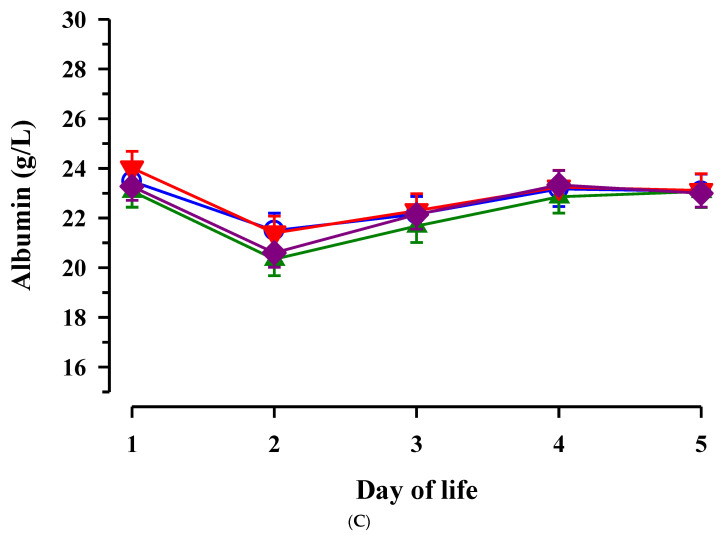
Plasma immunoglobulin G (**A**), total protein (**B**) and albumin (**C**) concentrations in calves dependent on maternal fatty acid supplementation (○ control, CON *n* = 9; ▲ EFA, essential fatty acids, *n* = 9; ▼, CLA, conjugated linoleic acid, *n* = 9; ♦, EFA + CLA, *n* = 10) and time after birth. Day 1 corresponded to the day of birth. ^ab^ Different superscripts mark significant differences between groups at similar time points (*p* < 0.05). ^Y^ between calves with and without maternal CLA supplementation (*p* < 0.05).

Plasma IgG concentrations increased from day 1 to day 2 of life in all calves (*p* < 0.001) and remained elevated until the end of the study (Figure 2A). Total protein concentrations increased from day 1–2 in all calves (*p* < 0.001; Figure 2B). Total protein tended to be elevated due to maternal CLA treatment (*p* = 0.072) and after birth tended to increase faster in CLA than non-CLA calves (CLA x time interaction, *p* = 0.090). Total protein was higher on day 2 of life in CLA and EFA + CLA than in EFA calves (*p* < 0.05). Plasma albumin concentrations in plasma decreased independently from maternal fatty acid supplementation from day 1 to day 2 of life (*p* < 0.001) and increased thereafter until day 4 (*p* < 0.05; Figure 2C).

**Figure 3:**
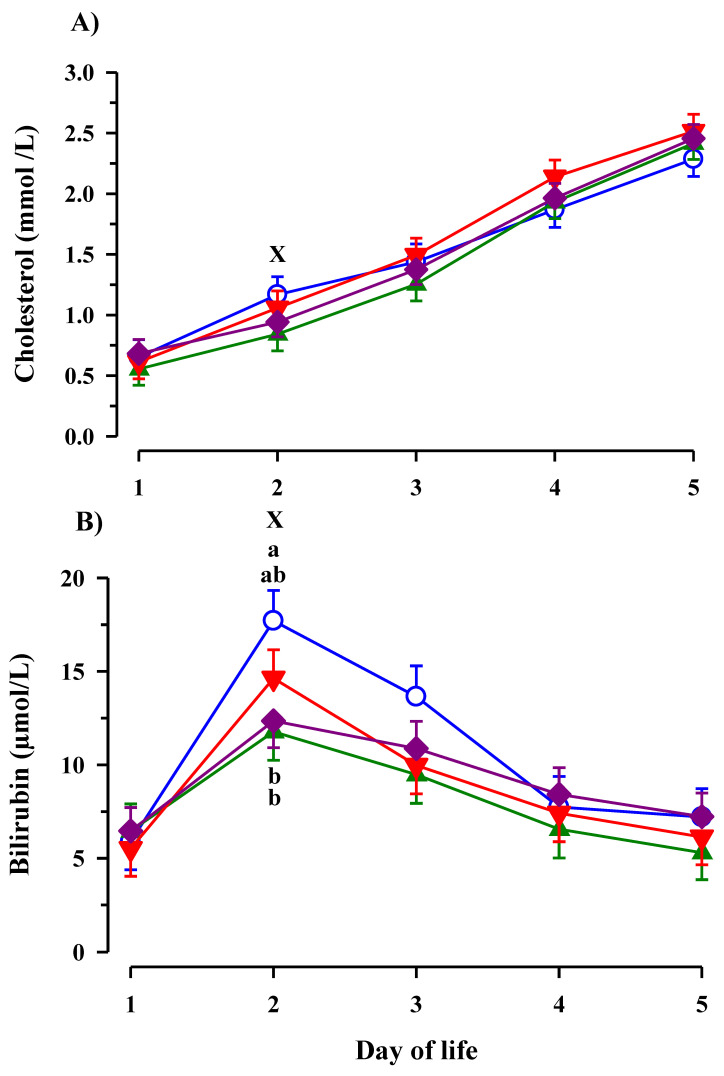
Plasma cholesterol (**A**) and bilirubin (**B**) concentration in calves dependent on maternal fatty acid supplementation (○ control, CON *n* = 9; ▲ EFA, essential fatty acids, *n* = 9; ▼, CLA, conjugated linoleic acid, *n* = 9; ♦, EFA + CLA, *n* = 11) and time after birth. Day 1 corresponded to the day of birth. ^ab^ Different superscripts mark significant differences between groups at similar time points (*p* < 0.05). ^X^ designates significant differences between calves with maternal EFA supplementation and without maternal EFA supplementation within a time point (*p* < 0.05).

Plasma cholesterol concentration increased (*p* < 0.001) from d 1 to d 5 of life, whereas plasma total bilirubin increased (*p* < 0.001) from day 1 to day 2 of life and decreased (*p* < 0.001) thereafter (Figure 3A,B). The increase of bilirubin was much more pronounced in CON calves leading to a significantly higher bilirubin concentration in CON calves compared to EFA and EFA + CLA calves on day 2 of life (*p* < 0.05). On d 2, plasma total bilirubin (*p* < 0.01) and cholesterol (*p* = 0.04) were lower in calves with maternal EFA-supplementation than in the calves whose dams did not receive EFA.

**Figure 4:**
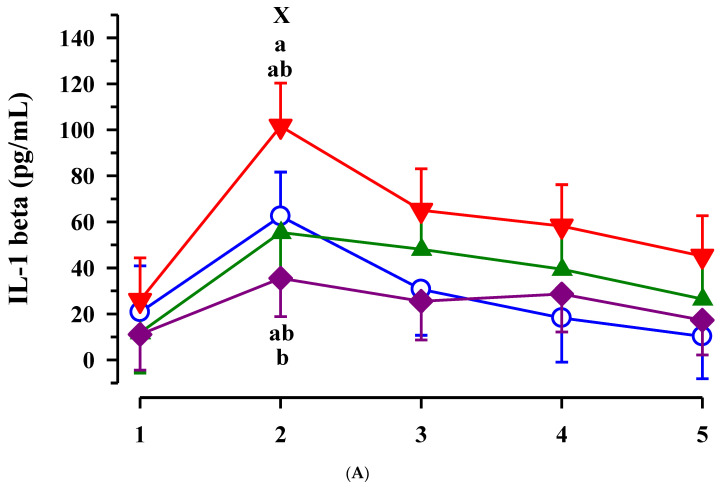

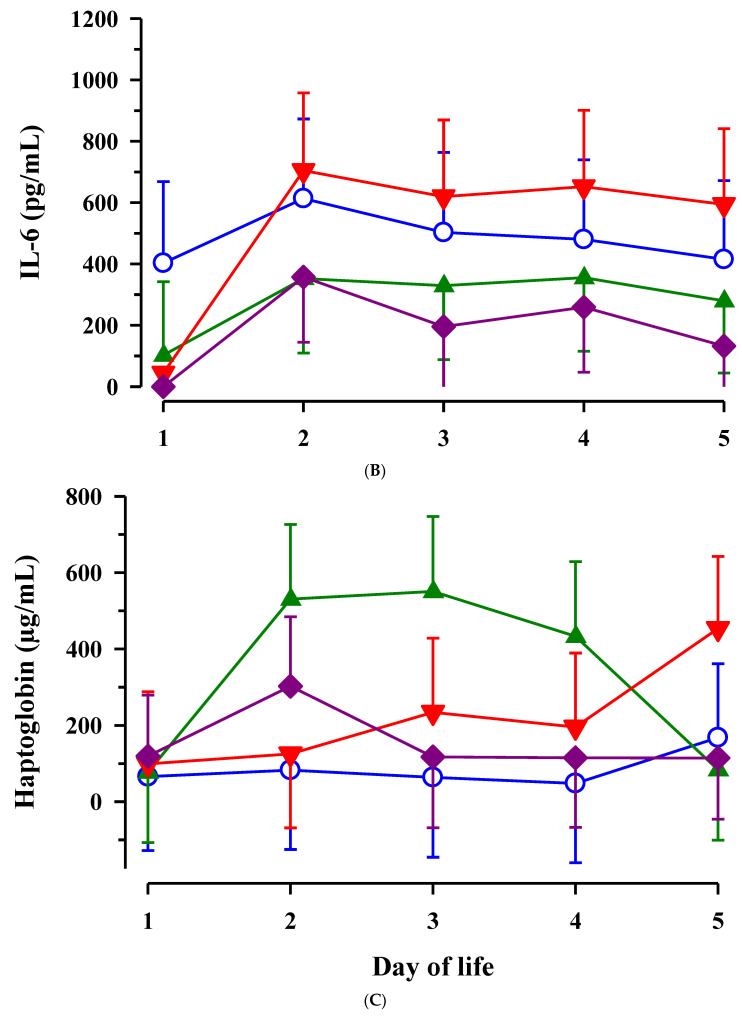
Plasma interleukin (IL) 1-β (**A**), IL-6 (**B**) and haptoglobin (**C**) concentration in calves dependent on maternal fatty acid supplementation (○ control, CON *n* = 9; ▲ EFA, essential fatty acids, *n* = 9; ▼, CLA, conjugated linoleic acid, *n* = 9; ♦, EFA + CLA, *n* = 11) and time after birth. Day 1 corresponded to the day of birth. ^ab^ Different superscripts mark significant differences between groups at similar time points (*p* < 0.05). ^X^ designates significant differences between calves with maternal EFA supplementation and without maternal EFA supplementation within a time point (*p* < 0.05).

While the plasma interleukin 1-β concentration in CLA calves increased from day 1 to day 2 of life (*p* < 0.001), the concentrations showed only marginal alterations in the other groups (Figure 4A). Consequently, CLA calves had higher interleukin 1-β concentration compared to EFA + CLA calves on day 2 of life (*p* = 0.009). Furthermore, plasma interleukin 1-β was lower in calves with than without maternal EFA treatment on day 2 of life (*p* = 0.017). Interleukin 1-β correlated with the bilirubin concentration in plasma of calves (r = 0.263; *p* = 0.001; Table 3). Plasma haptoglobin concentration indicated a trend for an EFA x CLA (*p* = 0.091) and EFA x time (*p* = 0.086) interaction, respectively, and tended to increase during the first 24 h of life in calves with maternal EFA treatment (*p* = 0.08; Figure 3C). On day 2 of life, plasma haptoglobin tended to be higher in calves with than without EFA treatment (*p* = 0.056).

Considering calves from dams treated with CLA, interleukin 6 was affected in a time dependent manner similar to interleukin 1-β (*p* < 0.001); however, there were no significant differences between groups within time points (Figure 4B).

**Figure 5:**
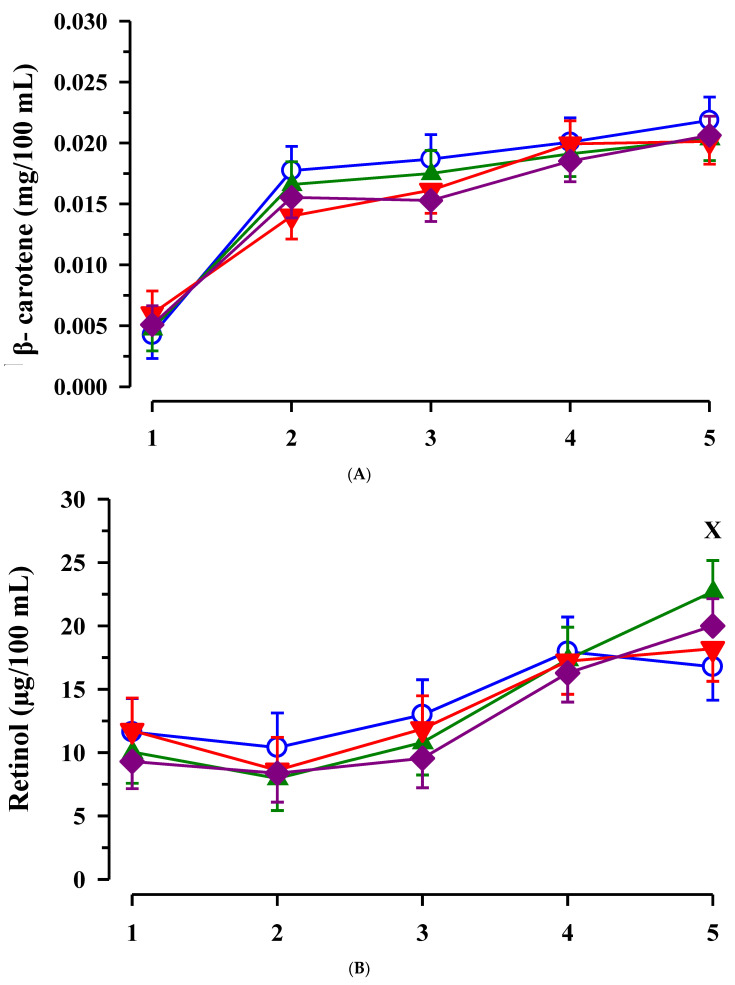

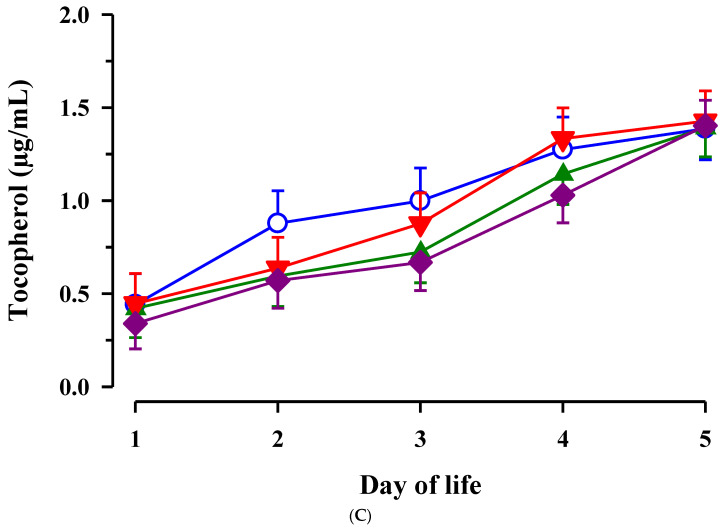
Plasma β- carotene (**A**), retinol (**B**), and tocopherol (**C**) concentrations in calves dependent on maternal fatty acid supplementation (○ control, CON *n* = 9; ▲ EFA, essential fatty acid, *n* = 9; ▼, CLA, conjugated linoleic acid, *n* = 9; ♦, EFA + CLA, *n* = 11) and time after birth. Day 1 corresponded to the day of birth. ^X^ marks differences between calves with maternal EFA supplementation and without maternal EFA supplementation within the time points (*p* < 0.05).

The plasma concentration of β- carotene increased from day 1 to day 5 of life in all calves (*p* < 0.001; Figure 5A). A trend for a CLA-by-day interaction was detected for β- carotene concentration (*p* = 0.083), but there were no individual treatment effects in certain time points. Plasma retinol concentration increased in a time dependent manner (*p* < 0.001) and concentration on day 5 of life was higher in calves from dams treated with EFA than in calves without maternal EFA treatment (*p* = 0.048; Figure 5B).

The plasma concentration of tocopherol increased after birth until the end of the study (*p* < 0.001), but showed no differences between groups (Figure 5C).

**Figure 6:**
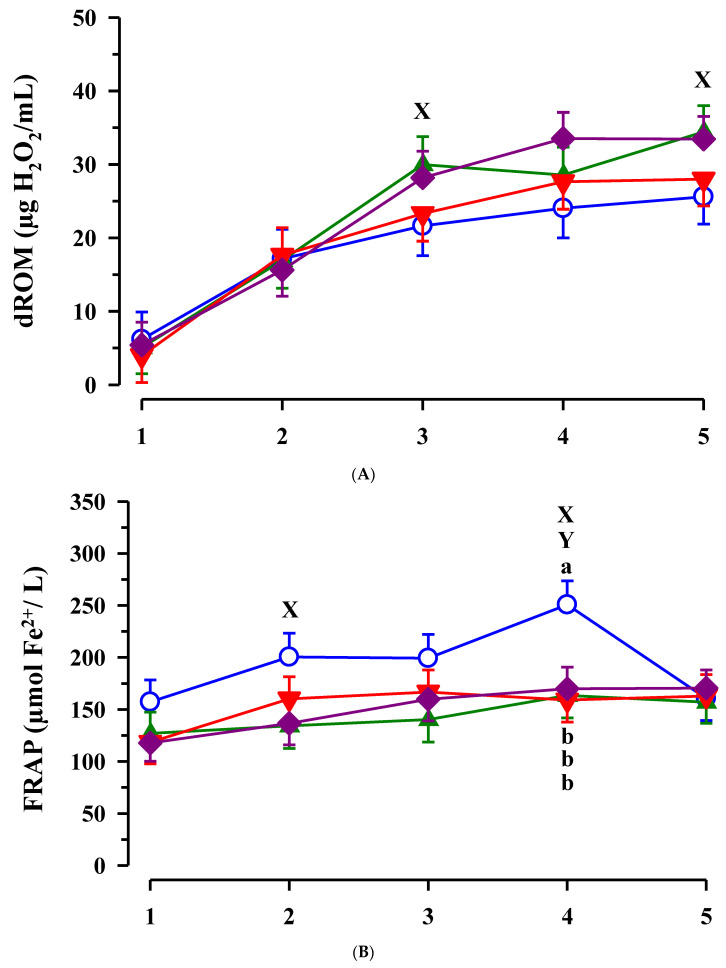

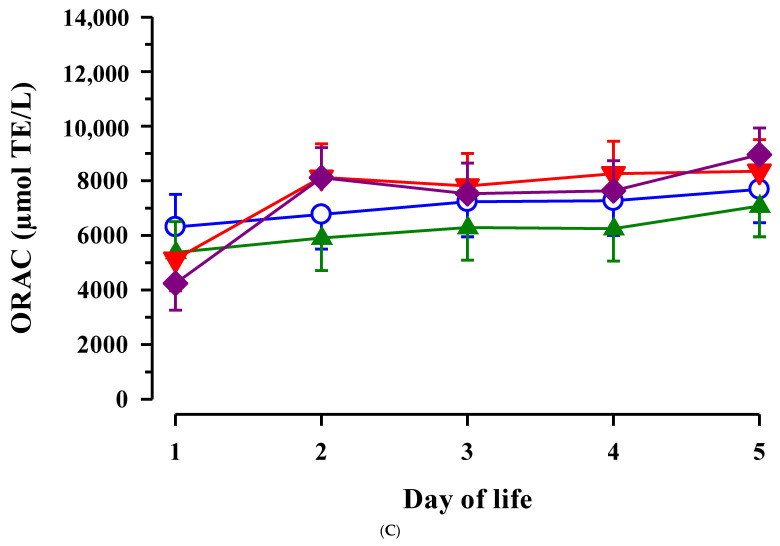
Plasma derivates of reactive oxygen metabolites (dROM) (**A**), ferric ion reducing anti-oxygen power (FRAP) (**B**) and oxygen radical absorbance capacity (ORAC) (**C**) in calves dependent on maternal fatty acid supplementation (○ control, CON *n* = 9; ▲ EFA, essential fatty acids, *n* = 9; ▼, CLA, conjugated linoleic acid, *n* = 9; ♦, EFA + CLA, *n* = 11) and time after birth. Day 1 corresponded to the day of birth. ^ab^ Different superscripts mark differences between groups at similar time points (*p* < 0.05). ^X^ designates significant differences between calves with maternal EFA supplementation and without maternal EFA supplementation at similar time points and ^Y^ between calves with and without maternal CLA supplementation (*p* < 0.05).

The concentration of dROM was significantly increased from day 1 to day 5 of age (*p* < 0.001; Figure 6A). On day 3 and 5 of life, plasma dROM was higher in calves with than without maternal EFA supplementation (*p* < 0.05). dROM was positively correlated with concentrations of vitamins in the plasma (r > 0.338; *p* < 0.05; Table 3).

FRAP was influenced by time (*p* < 0.001) and increased from day 1 to day 4 of life in CON animals (*p* < 0.05; Figure 6B). A significant EFA effect on FRAP was detected (*p* = 0.007), but also a significant EFA-by-CLA interaction (*p* = 0.009). Additionally, calves with maternal EFA supplementation had lower plasma FRAP on day 2 of life (*p* = 0.014) and tended to be lower on day 3 of life in these calves compared to calves without maternal EFA supplementation (*p* = 0.071). On day 4 of life, CON calves showed a higher FRAP compared to all other groups (*p* < 0.05). At the same time point, FRAP was lowered by maternal EFA and by CLA treatment (*p* = 0.037 and *p* = 0.019). There was a positive correlation between FRAP and bilirubin concentrations in plasma on day 2 of life (r = 0.637; *p* < 0.001).

The ORAC was higher in EFA + CLA calves on day 2 and day 5 of life compared to the initial level (*p* < 0.05) (Figure 6C). On day 2 of life plasma ORAC tended to be higher in calves with maternal CLA supplementation compared to calves without maternal CLA supplementation (*p* = 0.070) leading in a significant CLA-by-time interaction (*p* = 0.050).

## 4. Discussion

In a companion paper of Uken et al. [26] the effects of the maternal fatty acid supplementation on the colostrum and milk composition of the dams from the calves used in the present study were demonstrated. The results indicated lower dry matter content in the first colostrum milking after calving of CLA- than non-CLA-treated cows and lower protein concentration in the first colostrum of CLA-treated cows. Lower protein concentrations seemed to be not related with the immunoglobulin concentrations in the milk presented in the current publication. EFA treatment resulted in lower fat concentrations in colostrum of EFA supplemented cows but CLA did not reduce milk fat during first milkings after calving [26].

Plasma Ig and total protein concentrations in calves reflected the Ig concentrations in colostrum of their dams and indicated the absorption of Ig from colostrum and successful passive immunization of calves in the first days of life [5,34]. The maternal diet can influence the Ig concentration in colostrum of sows [15] and the relationship between neonatal absorption of polyunsaturated fatty acid and Ig was recently discussed in piglets [35]. The maternal EFA and CLA supplementation in the present study affected the EFA and CLA status in the calves [26] but did not influence the total IgG absorption in calves. Recent studies in calves showed positive as well as negative effects of maternal EFA supply on neonatal IgG absorption [25,36]. The lower IgG_2_ concentration in the transition milk of the 2nd day in EFA cows might not explain the differences in plasma total protein concentration, as total plasma IgG on day 2 after birth was not different among groups. Plasma albumin and haptoglobin concentrations, which are also an integral part of total proteins, were not a plausible explanation for the lower total protein concentration in EFA calves because of no or opposite influences of the fatty acid treatments on these plasma traits in the calves. Therefore, we currently cannot explain the up to 10 g/L lower plasma concentration of total protein in the EFA calves compared with the other groups on d 2 of life and we cannot exclude other causes such as an altered protein accretion. However, because of the slightly higher haptoglobin concentrations in EFA calves compared to CON calves we speculated that lower plasma total protein concentrations in EFA calves might indicate an altered liver activity.

In contrast to studies of Panousis et al. [37], the current study revealed no differences between hematological traits of male and female calves. Nevertheless, the values of calves in the present study were in the reference intervals described by Knowles et al. [38], Brun-Hansen et al. [39] and Panousis et al. [37], except for hemoglobin, RDW, MCHC and thrombocytes, which were slightly lower in the mentioned studies. The age-dependent effects on HCT, hemoglobin, RDW, erythrocyte cell counts and MCV were also described in studies of Knowles et al. [38] and Panousis et al. [37]. In both studies, it was demonstrated that leucocyte cell counts decrease until day 3 or 4 of life. In comparison with the present results, it might be assumed that EFA supports the period of physiological decrease of leucocyte counts after birth while CLA shortened or inhibited this period. The biological relevance of this aspect has to be studied in further trials. According to Knowles et al. [38] proportions of lymphocytes increase physiologically in calves after birth but proportions of neutrophils decrease. Interestingly, only in EFA calves there was a trend for this physiological increase of lymphocyte proportions on day 5 of life. In contrast, the combined EFA + CLA treatment seemed to intensify the decrease of the lymphocyte proportion, leading to the assumption that other leucocyte populations increase such as neutrophilic granulocytes. Because only proportions of basophilic granulocytes were additionally determined we can only exclude a marked increase of this cell population in the EFA + CLA calves. Essential fatty acids such as linoleic acid are able to increase the mitogen-stimulated proliferation of PBMCs at lower concentrations in cattle [18,40]. At higher concentrations, this fatty acid inhibited mitogen-stimulated PBMC proliferation but also unstimulated lymphocyte proliferation [18,40].

The continuous increase of plasma cholesterol in the calves was in accordance with the increase in plasma cholesterol after parturition in dams [19]. The lower plasma cholesterol concentration on day 2 of life in the EFA calves was probably a consequence of lower milk fat content in the colostrum of the dams supplemented with EFA [26]. It might also reflect a lower liver activity in EFA calves. Furthermore, EFA might have cholesterol clearing effects via the activation of PPARα, which corresponds to lower cholesterol levels found previously in the plasma of calves supplemented with essential fatty acids [13,41].

The increase of bilirubin in the first 24 h after birth is a physiological phenomenon that has already been shown in the study of Kurz and Willet [42] with neonatal calves [42]. The increase from day 1 to day 2 of life might indicate an enhanced degradation of fetal erythrocytes and the increased synthesis of bilirubin in calves [43]. Regarding the lower postnatal increase in plasma bilirubin in the EFA calves, we speculate that maternal EFA treatment may decelerate erythrocyte decay in newborn calves, probably because of stabilization of the fetal erythrocyte membrane by EFA incorporation. The erythrocyte membranes of the dams were enriched, especially with α-linolenic acid and other n-3 FA, due to EFA supplementation [19]. However, the number of erythrocytes in calf blood did not differ among the groups. In addition, the plasma bilirubin concentration was lower at parturition in the dams treated with EFA only [19]. A decrease in the n-6/n-3 fatty acid ratio reduced the plasma bilirubin concentration and prevented liver diseases in preterm infants [44] and in neonatal piglets [45] fed parenteral nutrition. The stabilization of the erythrocyte membrane by reducing the n-6/n-3 fatty acid ratio in the membrane may lead to less iron release from fetal erythrocytes. Free iron is able to generate harmful oxygen species by producing oxidant hydroxyl radicals through the Fenton reaction [46,47]. Unfortunately, plasma iron was not measured in the present study but plasma FRAP was lower in EFA calves and there was a close correlation between plasma bilirubin and FRAP on day 2 of life.

In studies of Opgenorth et al. [48] it was shown that n-3 fatty acids supplemented by colostrum are able to improve the anti-inflammatory state of neonatal calves. In the present study the maternal EFA supplementation decreased and CLA increased the secretion of the pro-inflammatory cytokine interleukin 1-β on day 2 of life. Similarly, interleukin 6 production was increased in the CLA group with time, which might be the result of the increased interleukin 1-β concentration. Interleukin 1-β is able to induce the release of prostaglandin E2, which is a potent stimulator of interleukin 6 [49,50,51].

Plasma interleukin 1-β may have additionally contributed to the increased bilirubin plasma concentration in CLA calves on day 2 of life. Bilirubin is known as an inflammatory marker in cattle [52]. Elevated levels of bilirubin are toxic for the organism and have to be cleared rapidly, however, it was shown that bilirubin is also a potent anti-oxidant [53,54] and an effective inhibitor of inducible nitric oxide synthase (iNOS) [55]. This enzyme is known as a potent stimulus of reactive oxygen species production. The production of iNOS is upregulated by interleukin 1-β and iNOS are elevated in the liver and ileum 24 h after birth in calves [49,56]. An increase in the production of reactive oxygen species can lead to oxidative stress. Therefore, the upregulation of bilirubin in non-EFA calves might be a response and clearing mechanism of increased interleukin 1-β production and resulting increased reactive oxygen species release. However, according to dROM no differences in reactive oxygen metabolites in plasma were found between EFA and non-EFA calves. The anti-oxidative potential of bilirubin might be supported by the significant correlation between bilirubin and FRAP on day 2 of life and might be an explanation for the higher FRAP of non-EFA groups compared to EFA groups on this day. Relationships between FRAP and bilirubin were already shown in rats [57].

Whether the increase of interleukins is a response to increased bilirubin concentrations because of its toxicity which was possibly induced by erythrocyte degradation or whether this is a clearing mechanism of the enhanced pro-inflammatory interleukin release might be investigated in further studies. However, in the present study clear relations between these factors were shown and that these relationships depended on the maternal fatty acid supply which in turn altered the colostral and plasma fatty acid composition of the offspring [26,27]. Wiedemann et al. [54] found relationships between the plasma oxidation rate and bilirubin as well as the polyunsaturated fatty acid concentrations in plasma of newborns. They reported improved protection against oxidation immediately after birth by higher bilirubin but lower polyunsaturated fatty acid concentration. Compared to bilirubin, other measured plasma anti-oxidants increased more slowly after birth which might be an evidence of the importance of bilirubin especially during the first hours after birth. With regard to these aspects, CLA and CON calves might be superior compared to EFA calves because of the provoked increase of bilirubin concentrations. Indeed, in the case of EFA, plasma FRAP was reduced on day 2, which corresponded to the increased dROM plasma concentration on day 3 of life. In studies of Ballou and DePeters [14], n-3 fatty acid supplementation resulted in a higher percentage of oxidative burst-producing polymorphonuclear leucocytes in calves.

Similar to the oxidative system as indicated by dROM, the anti-oxidative blood components such as β- carotene, retinol and tocopherol, FRAP and ORAC increased age-dependently after birth in all calves. Boosting of the anti-oxidative system in calves after birth leading to a better protection against oxidative stress in this period compared to cows was also demonstrated by Gaál et al. [58]. Because the oxidative and anti-oxidative status of calves did not differ immediately after birth, it was suggested that treatment-dependent differences between calves where more related to colostral supply. Abuelo et al. [2,59] pointed at the importance of the redox balance of the colostrum on the oxidative status of the calf. Blum et al. [60] already demonstrated the importance of colostrum on the vitamin status of newborn calves and emphasized the relationships between the absorption of fatty acids and fat-soluble vitamins such as retinol. According to the present results, the fatty acid composition in colostrum, which was influenced by the maternal fatty acid supply, did not influence the absorption of fat-soluble vitamins few days after birth in a consistent manner. An elevated retinol plasma concentration was found with respect to EFA treatment on day 5 of life, which might have boosted the anti-oxidant status in these calves. Correlations between dROM and plasma vitamins indicate their role to balance the age-dependent increases in dROM concentrations. In turn, EFA calves might have a more efficient protection against pathogens by increased radical production and similarly improved anti-oxidative status, which prevents against oxidative stress in a later stage after birth. Additionally, in studies of Opgenorth et al. [61], n-3 fatty acid supplementation by colostrum improved the oxygen status of newborn calves.

## 5. Conclusions

The maternal fatty acid supplementation influenced the inflammatory response shortly after birth. Especially, EFA might have beneficial effects on the prevention of inflammation after birth and against oxidative stress in a later stage after birth. Furthermore, EFA reduced the plasma bilirubin concentrations in an early period. CLA and saturated fatty acids might increase the inflammatory response. Similarly, bilirubin was increased by this maternal supplementation, which might serve as a protector against oxidative stress in this early stage. In general, calves have potent instruments for the clearance of inflammatory or oxidative stress after birth in which bilirubin seemed to be involved. In further studies, the relationships between bilirubin and the interleukin response as well as the anti-oxidative and the oxidative status in the neonatal calf should be studied in more detail to support the present findings.

## Figures and Tables

**Table 1:** Experimental design and animals.

Group	Maternal Fatty Acid Supplementation (MFAS) *	Number of Calves (*n*)	Calf Sex	
			Male	Female
Control (CON)	76 g/day coconut oil ^†^	9	5	4
EFA	78 g/day linseed oil ^‡^ and 4 g/day safflower oil ^⸸^	9	4	5
CLA	38 g/day Lutalin ^§^ (CLA; *cis*-9, *trans*-11, *trans*-10, *cis*-12 CLA, 10 g/day each)	9	1	8
EFA + CLA	78 g/day linseed oil + 4 g/day safflower oil + 38 g/day Lutalin	11 ^$^	4	7

* abomasal supplementation of dams starting 63 days before expected calving until early lactation, amounts of supplement were halved during dry period starting 6 weeks before calving; ^†^ Bio-Kokosöl #665, Kräuterhaus Sanct Bernhard KG, Bad Ditzenbach, Germany; ^‡^ DERBY Leinöl #4026921003087, DERBY Spezialfutter GmbH, Münster, Germany; ^⸸^ GEFRO Distelöl, GEFRO Reformversand Frommlet KG, Memmingen, Germany; ^§^ BASF SE, Ludwigshafen, Germany; ^$^ includes 9 single born calves and one twin pair.

**Table 2:** Hematology of calves depending on maternal fatty acid supplementation (MFAS) and time after birth.

Variable	Time ^#^	MFAS				*p*-Values					
		CON ^α^	EFA ^β^	CLA ^γ^	EFA + CLA	EFA	CLA	EFA × CLA	Time	EFA × Time	CLA × Time
Hematocrit, %	1	30.9 ± 3.8	26.6 ± 3.6	30.1 ± 3.7	27.1 ± 3.2	0.21	0.95	0.38	<0.001	0.37	0.49
	5	23.0 ± 3.6 ***	19.7 ± 3.3 ***	23.0 ± 3.5 ***	20.6 ± 2.9 ***						
Hemoglobin, g/dL	1	8.48 ±1.01	7.31 ± 0.94	8.47 ± 0.97	7.57 ± 0.83	0.17	0.81	0.85	<0.001	0.44	0.81
	5	6.64 ± 0.95 ***	5.69 ± 0.87 ***	6.72 ± 0.91 ***	5.98 ± 0.77 ***						
Erythrocytes, 10^6^/mm^3^	1	7.20 ± 0.77	6.54 ± 0.72	7.37 ± 0.75	6.66 ± 0.63	0.22	0.73	0.98	<0.001	0.85	0.78
	5	5.77 ± 0.75 ***	5.16 ± 0.69 ***	5.92 ± 0.72 ***	5.39 ± 0.60 ***						
RDW ^†^, %	1	16.9 ± 0.6	16.7 ± 0.6	15.8 ± 0.6	16.8 ± 0.5	0.49	0.19	0.12	0.05	0.19	0.55
	5	16.9 ± 0.7	16.4 ± 0.6	15.6 ± 0.6	16.5 ± 0.6						
MCV ^‡^, µm^3^	1	42.8 ± 1.6	41.2 ± 1.6	40.9 ± 1.6	41.0 ± 1.4	0.72	0.45	0.47	<0.001	0.050	0.29
	5	40.2 ± 1.5 ***	39.2 ± 1.4 ***	38.6 ± 1.5 ***	39.3 ± 1.3 ***						
MCH ^⸸^, pg	1	11.8 ± 0.4	11.4 ± 0.4	11.5 ± 0.4	11.5 ± 0.3	0.36	0.71	0.37	0.016	0.62	0.82
	5	11.7 ± 0.4	11.2 ± 0.3	11.4 ± 0.3	11.3 ± 0.3						
MCHC ^§^, g/dL	1	27.5 ± 0.6	27.8 ± 0.6	28.5 ± 0.6	27.9 ± 0.5	0.54	0.25	0.21	<0.001	0.33	0.91
	5	28.7 ± 0.7 ***	29.0 ± 0.7 ***	29.9 ± 0.7 ***	28.8 ± 0.6 ***						
Leucocytes, 10^3^/mm^3^	1	9.4 ± 1.4	8.9 ± 1.4	10.8 ± 1.4	9.4 ± 1.2	0.029	0.14	0.44	0.01	0.09	0.61
	5	8.4 ± 1.4 ^ab^	6.2 ± 1.3 ^b^	10.7 ± 1.4 ^a^	6.9 ± 1.2 ^b^						
Lymphocytes, %	1	34.3 ± 8.3	33.0 ± 7.9	35.2 ± 8.0	42.6 ± 6.9	0.44	0.84	0.98	0.002	0.68	0.029
	5	25.6 ± 8.8	35.1 ± 8.1	22.5 ± 8.3	23.7 ± 7.1 *						
Atypical Lymphocytes, %	1	4.29 ± 0.54	4.19 ± 0.52	4.05 ± 0.52	3.90 ± 0.46	0.80	0.95	0.74	<0.001	0.85	0.27
	5	2.30 ± 0.57 *	2.04 ± 0.53 **	2.38 ± 0.53 *	2.57 ± 0.46						
Basophile Granulocytes, %	1	0.21 ± 0.17	0.10 ± 0.17	0.21 ± 0.17	0.47 ± 0.15	0.81	0.67	0.44	<0.001	0.25	0.09
	5	0.90 ± 0.16 **	0.82 ± 0.15 **	0.83 ± 0.15 *	0.67 ± 0.13						
Thrombocytes, 10^3^/mm^3^	1	482 ± 99	471 ± 93	645 ± 95	586 ± 82	0.49	0.13	0.50	0.33	0.76	0.18
	5	486 ± 103	490 ± 95	598 ± 98	486 ± 83						
MTV ^$^, µm^3^	1	6.36 ± 0.51	6.30 ± 0.47	6.80 ± 0.49	6.68 ± 0.42	0.59	0.36	0.85	0.052	0.29	0.27
	5	6.71 ± 0.52	6.52 ± 0.48	7.01 ± 0.50	6.63 ± 0.42						
Thrombocrit, %	1	0.32 ± 0.10	0.36 ± 0.09	0.48 ± 0.10	0.39 ± 0.08	0.54	0.24	0.36	0.14	0.26	0.32
	5	0.33 ± 0.11	0.33 ± 0.10	0.45 ± 0.10	0.33 ± 0.09						
Immature cells, %	1	25.4 ± 11.7	17.1 ± 11.8	16.6 ± 11.5	3.5 ± 11.8	0.16	0.68	0.77	0.55	0.78	0.12
	5	16.2 ± 8.5	2.6 ± 7.8	16.2 ± 7.9	14.6 ± 6.7						

^α^ CON, control; ^β^ EFA, essential fatty acids; ^γ^ CLA, conjugated linoleic acid; ^†^ RDW, red cell distribution width; ^‡^ MCV, mean corpuscular volume of erythrocytes; ^⸸^ MCH, mean corpuscular hemoglobin of erythrocytes; MCHC ^§^, mean corpuscular hemoglobin concentration of erythrocytes; MTV ^$^, mean thrombocyte volume; ^#^ Day of life; ^ab^ Different superscripts mark significant differences between groups (*p* < 0.05). * Asterisk show significant differences between days of life within groups (* *p* < 0.05; ** *p* < 0.01; *** *p* < 0.001).

**Table 3:** Correlations between selected plasma variables.

Variable	Correlated Parameters	Correlation Coefficient (*r*)	*p*-Value
Plasma traits			
Ig ^†^ G	Bilirubin	0.255	<0.001
	Interleukin 6	0.278	0.001
	β- carotene	0.447	<0.001
	Tocopherol	0.245	0.001
	dROM	0.491	<0.001
	ORAC ^§^	0.378	<0.001
Bilirubin	Interleukin 1-β	0.263	0.001
	Retinol	−0.388	<0.001
Interleukin 6	ORAC	0.270	0.001
β- carotene	Retinol	0.371	<0.001
	Tocopherol	0.648	<0.001
	dROM	0.610	<0.001
	ORAC	0.155	0.047
Retinol	Tocopherol	0.602	<0.001
	dROM	0.338	<0.001
Tocopherol	dROM	0.520	<0.001
dROM ^‡^	ORAC	0.238	0.002
FRAP ^⸸^	ORAC	0.245	0.002

^†^ Ig, immunoglobulin; ^‡^ dROM, derivates of reactive oxygen metabolites; ^⸸^ FRAP, ferric ion reducing anti-oxygen power; ORAC ^§^, oxygen radical absorbance capacity.

## Data Availability

The raw data supporting the conclusions of this article will be made available by the authors, without undue reservation.
